# Internal Aspiration in Macular Hole Surgery: A Retrospective Comparative Study

**DOI:** 10.1155/joph/7026243

**Published:** 2026-11-01

**Authors:** Ke Xu, Yanfeng Liao, Dan Li, Shuang Cui, Xuefeng Feng, Changguan Wang, Huijin Chen

**Affiliations:** ^1^ Beijing Key Laboratory of Restoration of Damaged Ocular Nerve Department of Ophthalmology Peking University Third Hospital Beijing China puh3.net.cn

**Keywords:** aspiration, best-corrected visual acuity (BCVA), ellipsoid zone, fluid inside the hole, full-thickness macular hole (FTMH)

## Abstract

**Purpose:**

To compare the structural and functional outcomes of pars plana vitrectomy (PPV) with or without aspiration of fluid from inside the macular hole for primary full‐thickness macular holes (FTMHs).

**Methods:**

We retrospectively analyzed the hole closure rate, the integrity of the ellipsoid zone (EZ), central retinal thickness (CRT), and best‐corrected visual acuity (BCVA) from 101 eyes of 101 patients with primary medium‐to‐large FTMH who underwent PPV with aspiration or nonaspiration technique within 1 month.

**Results:**

At 1 month postoperatively, Type 1 closure rates were 81.4% (48/59) in the aspiration cohort and 83.3% (35/42) in the non‐aspiration cohort; U‐type closure rates were 49.2% (29/59) and 52.4% (22/42), respectively. After the 1‐month visit, 61 of the 101 eyes (60.4%) were lost to follow‐up; the remaining 40 eyes (24 in the aspiration cohort and 16 in the nonaspiration cohort) were also analyzed at 3–6 months postoperatively. At 3–6 months postoperatively, Type 1 closure rates were 75.0% (18/24) in the aspiration cohort and 87.5% (14/16) in the non‐aspiration cohort; U‐type closure rates were 58.3% (14/24) and 62.5% (10/16), respectively. No significant between‐cohort differences were found in the unadjusted analyses; multivariable models likewise showed no significant association of aspiration with closure or BCVA. BCVA of the 40 eyes (Cohort 1 + Cohort 2) showed no significant change from baseline (1.00 logMAR [Q1–Q3: 0.92–1.40]) to 1 month postoperatively (1.05 logMAR [Q1–Q3: 0.92–1.49 logMAR]) and improved significantly at 3–6 months postoperatively (0.82 logMAR [Q1–Q3: 0.52–1.00 logMAR]) (*p* < 0.001), which was correlated with a significant decrease in the EZ defect from 1443.50 μm (Q1–Q3: 1053.50–1875.50 μm) at 1 month to 797.00 μm (Q1–Q3: 264.75–1610.00 μm) at 3–6 months (*p* = 0.003).

**Conclusion:**

Aspiration of fluid from inside the macular hole seems to provide no additional benefit in this retrospective study for the surgical treatment of medium‐to‐large primary FTMHs. Improvement in postoperative visual function occurs simultaneously with the reduction of EZ defects.

## 1. Introduction

A full‐thickness macular hole (FTMH) is defined as a foveal lesion with interruption of all retinal layers from the internal limiting membrane (ILM) to the retinal pigment epithelium (RPE) that can lead to severe central vision impairment [1–3]. The primary FTMH resulted from anomalous vitreomacular adhesion and traction. Surgical treatment of FTMH was first described by Kelly and Wendel in 1991 [4]. Thereafter, pars plana vitrectomy (PPV) has become the standard treatment for macular hole (MH). The conventional techniques involve removing the residual posterior vitreous cortex, peeling the ILM, and using gas tamponade. However, the mechanisms of hole closure have not been fully elucidated, and the surgical outcomes of refractory MHs, such as cases with chronic or large MH, persistent or recurrent MH after previous surgery, and highly myopic MH, remain discouraging, leading to a variety of surgical technique modifications. These include methods providing scaffold or covering for cell proliferation and isolation from vitreous fluid, like inverted ILM flap technique [5], autologous blood [6], amniotic membrane plug [7], and autologous retina transplantation [8]. However, there is no consensus on these techniques, and maintaining the plugging material in place requires high surgical skills. The second is loosening and approximation of the edges of MH, promoting concentric closure of the hole [9–13]. Techniques include mechanical approximation and compression of the hole edges [9], tapping of MH edges [10], hydromassage or hydrodissection of MH edges [11, 12], and endodrainage with silicone oil tamponade [13]. Small case series have described aspiration of viscous fluid from inside the hole without direct contact with the hole edge or RPE, with the aim of flattening the fluid cuff and reducing resistance to closure under air or gas tamponade [14–17]. However, intrahole aspiration is not a standard step in MH surgery, and both its incremental benefit and its potential for tissue injury remain uncertain. In this retrospective case–control study, we aimed to compare the hole closure rate, foveal configuration, central retinal thickness (CRT), integrity of the ellipsoid zone (EZ), and best‐corrected visual acuity (BCVA) in patients undergoing PPV with or without aspiration of fluid from inside the MH.

## 2. Materials and Methods

### 2.1. Study Design and Participants

This retrospective, interventional case–control series included patients who underwent surgery for FTMH exceeding 250 μm at Peking University Third Hospital, Beijing, China, between January 2020 and January 2025. The 250‐μm threshold was selected based on the classification by the International Vitreomacular Traction Study Group, in which MHs less than 250 μm are defined as small holes [18]. This threshold was set based on the consideration that MHs smaller than 250 μm close more easily, so aspiration is not necessary and could cause potential damage to the edge of the hole; by contrast, MHs larger than 250 μm have a higher risk of nonclosure, therefore included in this study. Exclusion criteria were recurrent or secondary MH, prior or concurrent retinal detachment (including MH‐associated retinal detachment), previous vitrectomy, or ocular trauma, and high myopia (> 6.00 D); cataract was not an exclusion criterion. Demographic and ophthalmic data were extracted from patient files and the Hospital Information System database, including BCVA, intraocular pressure (IOP), slit‐lamp, and ophthalmoscopic examinations. Spectral‐domain optical coherence tomography (SD‐OCT) (Spectralis, Heidelberg Engineering, Heidelberg, Germany) confirmed the diagnosis in all patients. The minimum and base diameters of the hole were measured, and each hole was staged according to the SD‐OCT morphologic staging system proposed by Pecaku et al. [19]: Stage A, separation of the neurosensory retina from the RPE with well‐defined external limiting membrane (ELM), EZ, and interdigitation zone; Stage B, thickening of the EZ; Stage C, patchy (moth‐eaten) photoreceptor loss; and Stage D, severe or complete loss of the inner and outer segments with a bare ELM. All patients were examined at 1 month postoperatively. Some of them were able to follow the doctor’s advice and were also examined at 3–6 months postoperatively, with 3–6 months postoperatively serving as the final assessment endpoint. BCVA, slit‐lamp, ophthalmoscopic, and OCT examinations were recorded at each follow‐up. Complications were routinely assessed and recorded during follow‐up, including iatrogenic retinal breaks, retinal detachment, cataract progression, endophthalmitis, cystoid macular edema, and elevated IOP, defined as an IOP of ≥ 30 mmHg at any postoperative visit. The horizontal linear length of EZ defects at the fovea, CRT, and the closure rate were evaluated using OCT. All quantitative OCT measurements were performed by a single masked retinal specialist who was unaware of the surgical technique. CRT was obtained by automatic segmentation from the OCT’s built‐in software, and EZ defects were measured manually with appropriate scale calibration in ImageJ (National Institutes of Health, Bethesda, MD). BCVA was recorded as decimal and transformed to logMAR notation. Written informed consent was obtained before surgery from each patient. The study adhered to the tenets of the Declaration of Helsinki and was approved by the Medical Ethics Committee of Peking University Third Hospital (ethical approval number: IRB00006761‐M20250897).

### 2.2. Surgical Technique

All patients underwent standard three‐port vitrectomies (25‐gauge), independently performed by three senior vitreoretinal surgeons (Doctors A, B, and C), each of whom had more than 15 years of experience and had performed more than 10,000 PPVs. For phakic eyes with significant cataracts or patients aged over 60 years, phacoemulsification and intraocular lens implantation (Phaco + IOL) were routinely combined. After core vitrectomy, posterior vitreous detachment was performed to ensure that the posterior hyaloid was entirely removed. An epiretinal membrane was peeled, if present. The ILM was peeled at least 2 papillary diameters around the MH with the assistance of indocyanine green staining (ICG; approximately 0.2 mL ICG diluted in 5% glucose, which was a 1 mg/mL solution). ICG is the only available agent for ILM staining currently in China. To avoid retinal toxicity, ICG was rapidly cleared about 3–5 s after injection into the vitreous cavity to minimize intraocular exposure. In Cohort 1 (Doctors A and B), during fluid–air exchange, the flute needle’s soft tip was placed above and very close to the hole taking care not to touch the RPE or hole edge. Viscous fluid inside the hole was sucked via the flute needle by siphon effect. The surgeon intermittently controlled suction by blocking or releasing the small opening on the flute needle until the viscous intrahole fluid was completely removed, as judged by the absence of “sticky sign” resembling the thread formation of melted cheese [15]. Centripetal movement of the hole edge and reduction or closure of the hole were observed, and suction was then stopped. In Cohort 2 (Doctor C), during fluid–air exchange, no attempt was made to aspirate fluid from the hole. ILM flap techniques and other techniques facilitating hole closure were not used in either group. Tamponade was performed with sterile air or C3F8 gas (4%–10%), with the choice of agent and concentration left to the surgeon’s discretion. C3F8 was diluted with medical‐grade sterile air in a sterile closed system immediately before use. The patients were asked to maintain a face‐down position for 3–7 days postoperatively, depending on the surgeon’s discretion.

### 2.3. Classification of FTMH Closure in Postoperative Follow‐Up

According to inner retinal configuration–based classification [20], hole closure was classified into U‐type (normal foveal contour), V‐type (steep foveal contour), or W‐type (foveal defect of neurosensory retina). Based on Kang’s classification [21], hole closure was categorized as Type 1 (complete closure without foveal defect), Type 2 (flattened MH with RPE exposure and lack of macular continuity), or Type 3 (unclosed). Only eyes meeting the criteria of U‐type or Type 1 closure were counted as true closure when calculating the closure rates in this study. Assessment of the closure type was performed by two masked retinal specialists who were unaware of the surgical techniques. If the two specialists could not reach an agreement, a third specialist was consulted. Interobserver reproducibility was quantified using weighted Cohen’s kappa. There was agreement in 98 of 101 cases for the inner retinal configuration–based classification and in 99 of 101 cases for Kang’s classification, with interobserver kappa values of 0.911 and 0.889, respectively.

### 2.4. Statistical Analysis

Statistical analyses were conducted using SPSS software (Version 27, IBM Corp., Chicago, IL, USA) and R Version 4.4.3 (R Foundation for Statistical Computing, Vienna, Austria). For continuous variables deviating from normal distribution, results were expressed as median with first (Q1) and third (Q3) quartiles; for variables following normal distribution, data were presented as mean ± standard deviation (SD). Categorical variables were reported as n (%). For independent‐group comparisons of continuous variables, Student’s t‐test was applied when both groups met normality assumptions; otherwise, the Mann–Whitney U test was used. Within‐group paired comparisons used the Wilcoxon signed‐rank test. Group differences in categorical data were evaluated using the chi‐square test or Fisher’s exact test. Correlation analyses used Spearman’s correlation coefficient (rho). Multivariable analyses used Firth penalized‐likelihood logistic regression (logistf package in R 4.4.3) for 1‐month U‐type and Type 1 closure and multiple linear regression for 1‐month and 3–6‐month BCVA, with postoperative BCVA as the outcome and preoperative BCVA as a covariate. The U‐type model included aspiration, age, and preoperative minimum hole diameter, whereas the Type 1 model included aspiration and minimum diameter because only 18 Type 1 nonclosures were available. Both BCVA models included aspiration, preoperative BCVA, minimum diameter, and age. Subgroup analyses were performed according to postoperative lens status and preoperative minimum hole diameter. Statistical significance was defined as *p* < 0.05.

## 3. Results

### 3.1. Demographic and Clinical Characteristics

Over five years, 101 patients (101 eyes) were enrolled (73 women and 28 men), with a median age of 65 years (Q1–Q3: 57.5–70 years). All 101 patients were evaluated at baseline and 1 month postoperatively; 40 also completed the 3–6‐month visit, whereas 61 did not. Table 1 shows the demographic and clinical characteristics of all 101 patients, and Table 2 shows those of the 40‐eye follow‐up subset. Eyes without 3–6‐month follow‐up differed from those completing follow‐up in stage of the MH, but not in treatment allocation, surgeon, or the other evaluated characteristics (Supporting Table S1). An iatrogenic retinal break occurred intraoperatively in one eye in each cohort (1/59 vs. 1/42); both were managed with photocoagulation during the same procedure and healed with no related sequelae recorded during follow‐up. A transient postoperative IOP elevation (32 mmHg) was recorded in one eye in the aspiration cohort and none in the nonaspiration cohort (1/59 [1.7%] vs. 0/42 [0%]); the IOP had returned to the normal range at the next follow‐up visit. No retinal detachment, endophthalmitis, or cystoid macular edema was recorded. One patient in Cohort 1 developed cataract 6 months postoperatively.

**TABLE 1:** Demographic and clinical characteristics of the 101 patients with 1‐month postoperative follow‐up.

	Total (*n* = 101)	Cohort 1 (*n* = 59)	Cohort 2 (*n* = 42)
Demographic characteristics			
Age (years), median (Q1–Q3)	65 (57.5–70)	65 (57–70)	66 (58–70)
Gender (female), *n* (%)	73 (72.3%)	38 (64.4%)	35 (83.3%)
Symptoms and ocular examinations			
Symptom duration (m), median (Q1–Q3)	6 (2–12)	6 (2–12)	6 (1–12)
BCVA (LogMAR), median (Q1–Q3)	1.00 (0.92–1.30)	1.10 (1.00–1.40)	1.00 (0.82–1.30)
IOP (mmHg), median (Q1–Q3)	14 (12–16)	14 (12–16)	14 (12–17)
Baseline pseudophakia, *n* (%)	6 (5.9%)	3 (5.1%)	3 (7.1%)
OCT evaluations of MH			
Minimum diameter (μm), median (Q1–Q3)	490.00 (364.85–613.50)	483.00 (367.00–710.00)	507.50 (340.25–612.25)
Basal diameter (μm), mean ± SD	1021.07 ± 306.34	1022.22 ± 307.35	1019.44 ± 308.62
With ERM, *n* (%)	30 (29.7%)	18 (30.5%)	12 (28.6%)
With operculum, *n* (%)	39 (38.6%)	23 (39.0%)	16 (38.1%)
Stage of the MH, *n* (%)			
Stage A	3 (3%)	0	3 (7.1%)
Stage B	69 (68.3%)	41 (69.5%)	28 (66.7%)
Stage C	26 (25.7%)	15 (25.4%)	11 (26.2%)
Stage D	3 (3%)	3 (5.1%)	0
Surgical details			
Combined Phaco + IOL, *n* (%)	82 (81.2%)	51 (86.4%)	31 (73.8%)
Choice of tamponade, *n* (%)			
Sterile air	62 (61.4%)	35 (59.3%)	27 (64.3%)
C_3_F_8_ gas	39 (38.6%)	24 (40.7%)	15 (35.7%)

*Note:* IOP, intraocular pressure. ERM, epiretinal membrane. Phaco + IOL, phacoemulsification and intraocular lens implantation.

Abbreviations: BCVA, best‐corrected visual acuity; MH, macular hole.

**TABLE 2:** Demographic and clinical characteristics of the 40 patients with both 1‐month and 3–6‐month postoperative follow‐up.

	Total (*n* = 40)	Cohort 1 (*n* = 24)	Cohort 2 (*n* = 16)
Demographic characteristics			
Age (years), median (Q1–Q3)	65 (54–70)	62 (49–68)	66 (55–70)
Gender (female), *n* (%)	27 (67.5%)	14 (58.3%)	13 (81.3%)
Symptoms and ocular examinations			
Symptom duration (m), median (Q1–Q3)	5 (1–12)	8 (1–12)	3 (1–12)
BCVA (LogMAR), median (Q1–Q3)	1.00 (0.92–1.40)	1.00 (0.94–1.40)	1.00 (0.85–1.37)
IOP (mmHg), median (Q1–Q3)	15 (12–17)	15 (12–17)	15 (13–18)
Baseline pseudophakia, *n* (%)	2 (5%)	1 (4.2%)	1 (6.3%)
OCT evaluations of MH			
Minimum diameter (μm), median (Q1–Q3)	521.50 (318.75–607.58)	487.00 (336.25–606.50)	554.00 (297.75–615.83)
Basal diameter (μm), mean ± SD	1058.22 ± 367.94	1071.00 ± 347.63	1039.04 ± 407.48
With ERM, *n* (%)	12 (30%)	7 (29.2%)	5 (31.3%)
With operculum, *n* (%)	17 (42.5%)	9 (37.5%)	8 (50.0%)
Stage of the MH, *n* (%)			
Stage A	2 (5%)	0	2 (12.5%)
Stage B	20 (50%)	15 (62.5%)	5 (31.3%)
Stage C	15 (37.5%)	6 (25%)	9 (56.3%)
Stage D	3 (7.5%)	3 (12.5%)	0
Surgical details			
Combined Phaco + IOL, *n* (%)	30 (75.0%)	20 (83.3%)	10 (62.5%)
Choice of tamponade, *n* (%)			
Sterile air	27 (67.5%)	14 (58.3%)	13 (81.3%)
C_3_F_8_ gas	13 (32.5%)	10 (41.7%)	3 (18.8%)

*Note:* IOP, intraocular pressure. ERM, epiretinal membrane. Phaco + IOL, phacoemulsification and intraocular lens implantation.

Abbreviations: BCVA, best‐corrected visual acuity; MH, macular hole.

### 3.2. Early Postoperative Outcomes

In 101 patients at 1 month postoperatively, there were no significant between‐cohort differences in BCVA, inner retinal configuration, Kang classification, CRT, or EZ defect (Supporting Table S2). Both cohorts showed no significant change in BCVA from baseline to 1 month postoperatively (Figure 1). Four patients in Cohort 1 and three in Cohort 2 underwent a second PPV after the 1‐month visit because of unsatisfactory hole closure, with no significant between‐cohort difference in reoperation rate. One‐month BCVA did not differ between cohorts in either the pseudophakic subgroup (1.00 vs. 0.96 logMAR, *p* = 0.740) or the phakic subgroup (*p* = 0.596). In the 40‐eye follow‐up subset, 3–6‐month BCVA was also similar between cohorts (pseudophakic subgroup, *p* = 0.889; phakic subgroup, *p* = 0.880) (Supporting Table S3). Closure rates did not differ between cohorts in the large‐hole subgroup (> 400 μm; U‐type, 40.5% vs. 40.7%, *p* = 0.983; Type 1, 73.8% vs. 77.8%, *p* = 0.709) or in the medium‐hole subgroup (≤ 400 μm; U‐type, *p* = 1.000; Type 1, *p* = 0.469), nor at 3–6 months in the 40‐eye follow‐up subset (Supporting Table S4).

**FIGURE 1:**
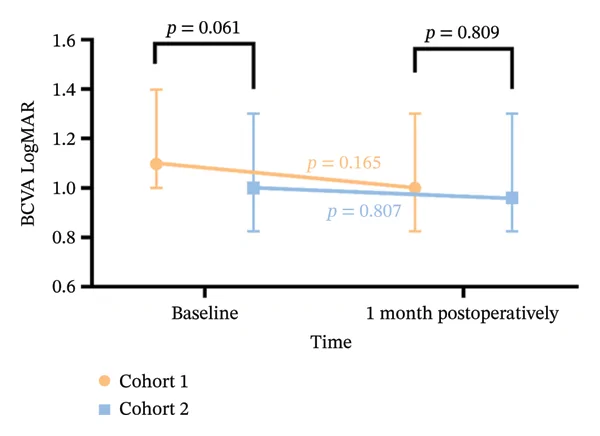
Best‐corrected visual acuity of the 101 eyes preoperatively and 1 month postoperatively.

### 3.3. Three to Six Months Follow‐Up Outcomes

At 3–6 months, among the 40 patients with follow‐up, U‐type closure rates were 58.3% in Cohort 1 and 62.5% in Cohort 2, and Type 1 closure rates were 75.0% and 87.5%, respectively. No significant difference existed in BCVA, hole closure rates, CRT, and EZ defects between Cohorts 1 and 2 at 1 month and 3–6 months postoperatively (Supporting Table S5, Figure 2). Among the patients who underwent second surgery for unclosed MH, three from Cohort 1 and two from Cohort 2 were in the 3–6‐month postoperative evaluations. Two patients from each cohort achieved Type 1 closure, and one patient in each cohort met the criteria for U‐type closure. No patients underwent second surgery between 3 and 6 months postoperatively.

**FIGURE 2:**
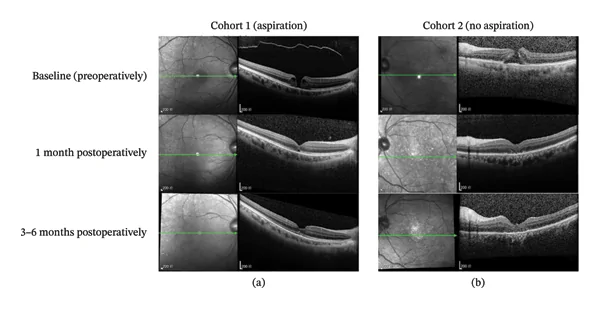
Optical coherence tomography (OCT) scans illustrating (a, b) macular foveal configuration in patients from Cohorts 1 and 2. Both show a U‐type and Type 1 closure at 1 month and at 3–6 months postoperatively.

### 3.4. Macular Structural and Visual Acuity Changes and Correlations

From 1 month to 3–6 months postoperatively, the U‐type closure rate increased from 45% to 60% (*p* = 0.179), and the Type 1 closure rate increased from 75% to 80% (*p* = 0.592); neither change was significant. The median EZ defect decreased from 1443.50 μm (Q1–Q3, 1053.50–1875.50 μm) to 797.00 μm (Q1–Q3, 264.75–1610.00 μm; *p* = 0.003), whereas median CRT did not change significantly (261.00 μm [Q1–Q3, 225.75–307.50 μm] vs. 246.50 μm [Q1–Q3, 223.50–286.50 μm]; *p* = 0.258). Median BCVA improved from 1.05 logMAR (Q1–Q3, 0.92–1.49) at 1 month to 0.82 logMAR (Q1–Q3, 0.52–1.00) at 3–6 months (*p* < 0.001); both Cohort 1 and Cohort 2 showed significant improvements (*p* = 0.002 and *p* = 0.006, respectively; Figure 3). Better BCVA correlated with U‐type closure and a smaller EZ defect but not with Type 1 closure or CRT (Supporting Table S6).

**FIGURE 3:**
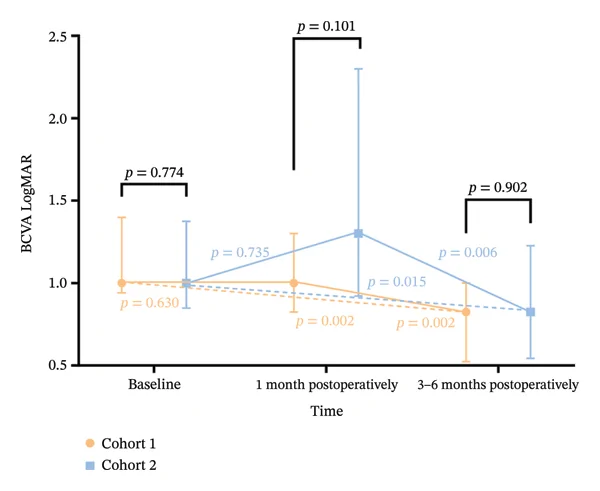
Best‐corrected visual acuity analysis of the 40 patients who had follow‐up results at 1 month and 3–6 months postoperatively.

### 3.5. Multivariable Analyses

Aspiration was not significantly associated with 1‐month U‐type closure after adjustment for age and preoperative minimum hole diameter (adjusted OR 0.89, 95% CI 0.37 to 2.11; *p* = 0.795), or with Type 1 closure after adjustment for preoperative minimum hole diameter (adjusted OR 0.93, 95% CI 0.30 to 2.82; *p* = 0.905). After adjustment for preoperative BCVA, minimum hole diameter, and age, the adjusted between‐cohort difference in BCVA (aspiration minus nonaspiration) was −0.094 logMAR at 1 month (95% CI −0.315 to 0.127; *p* = 0.400) and 0.044 logMAR at 3–6 months (95% CI −0.190 to 0.277; *p* = 0.706). Preoperative minimum hole diameter was consistently associated with both closure outcomes (*p* < 0.001) and with BCVA at 1 month (*p* = 0.012) and 3–6 months (*p* = 0.007). Complete model estimates are presented in Table 3, and the adjusted associations for aspiration and minimum hole diameter across the four models are summarized in Figure 4.

**TABLE 3:** Multivariable analyses of 1‐month macular hole closure and postoperative best‐corrected visual acuity.

A. Firth logistic regression analyses of 1‐month macular hole closure (*n* = 101)			
Outcome	Variable	Adjusted OR (95% CI)	*p* value
U‐type closure	Aspiration (vs. nonaspiration)	0.89 (0.37–2.11)	0.795
	Age, per 10‐year increase	0.88 (0.58–1.33)	0.525
	Minimum diameter, per 100‐μm increase	0.60 (0.45–0.77)	< 0.001

Type 1 closure	Aspiration (vs. nonaspiration)	0.93 (0.30–2.82)	0.905
	Minimum diameter, per 100‐μm increase	0.58 (0.41–0.77)	< 0.001

**B. Multiple linear regression analyses of postoperative BCVA**			

**Outcome**	**Variable**	**β (95% CI), LogMAR**	**p** **value**

1‐month BCVA (*n* = 101)	Aspiration (vs. nonaspiration)	−0.094 (−0.315, 0.127)	0.400
	Preoperative BCVA, per 0.1‐logMAR increase	0.002 (−0.021, 0.024)	0.892
	Minimum diameter, per 100‐μm increase	0.080 (0.018, 0.141)	0.012
	Age, per 10‐year increase	0.017 (−0.092, 0.125)	0.764

3–6‐month BCVA (*n* = 40)	Aspiration (vs. nonaspiration)	0.044 (−0.190, 0.277)	0.706
	Preoperative BCVA, per 0.1‐logMAR increase	0.038 (0.016, 0.059)	0.001
	Minimum diameter, per 100‐μm increase	0.082 (0.024, 0.140)	0.007
	Age, per 10‐year increase	0.074 (−0.015, 0.163)	0.101

*Note:* Odds ratios (ORs) and profile penalized‐likelihood 95% confidence intervals (CIs) were estimated using Firth logistic regression. *β* values are regression coefficients in logMAR units. *R* ^2^ was 0.079 for the 1‐month BCVA model and 0.445 for the 3–6‐month BCVA model.

Abbreviation: BCVA, best‐corrected visual acuity.

**FIGURE 4:**
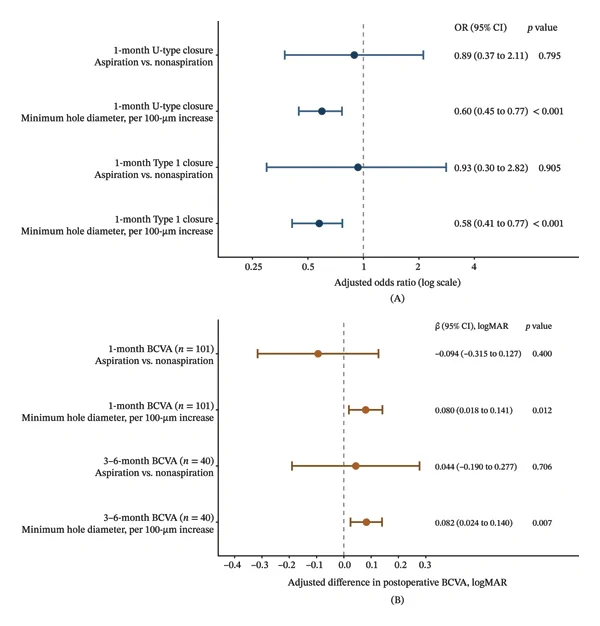
Multivariable analyses of 1‐month macular hole closure and postoperative best‐corrected visual acuity. (A) Adjusted associations with 1‐month closure (*n* = 101). (B) Adjusted associations with postoperative BCVA.

## 4. Discussion

Although the Gass classification remains used for classifying FTMH clinically, the International Vitreomacular Traction Study Classification System, an OCT‐based anatomic classification system, receives wider application [18]. Based on this system, medium‐to‐large MHs with aperture size > 250 μm were included in this study because surgical outcomes of the standard techniques for these MHs remain challenging. Conventional methods of MH surgery align with Tornambe’s hydration theory. The theory explains MH closure after successful surgery: A gas bubble covering the hole isolates the defect from liquid vitreous, halting vitreous fluid access to middle/outer macular tissue, allowing the RPE pump to remove intramacular fluid. As swelling resolves, the “drawbridge” closes, edges of the fovea reapproximate, and the hole disappears [22]. The hydration theory indicates that critical factors for hole closure are relieving traction and isolating the hole from vitreous fluid so that intraretinal fluid can be pumped out by RPE [22]. However, the hole closure rate using the conventional techniques alone is not so favorable; therefore, a variety of modifications in surgical techniques occur in recent years.

The hydration theory provided a rationale for considering fluid removal as an adjunct to conventional surgery [22]. Specifically, aspiration of viscous intrahole fluid may help to flatten the fluid cuff and reduce resistance to approximation of the hole edges [15, 16]. Shimada et al. described the viscous fluid during aspiration as showing a “sticky sign” resembling the thread formation of melted cheese, and their case series of 20 eyes with MH fluid aspiration showed 100% closure [15]. Ahmed Khan et al. [16] postulated that viscous fluid within the hole may delay flattening of MH, and the aspiration may result in flattening of the cuff area and reduction in the resistance to closure with a gas or air tamponade overlying the hole, which reached a closure rate of 97.5% in their case series of 40 eyes. Ohana et al. [14] reported that aspiration performed under perfluorocarbon liquid (PFCL) allowed more controlled removal of intrahole fluid, while providing retinal stabilization and minimizing retinal deformation. However, the high closure rates in these small, uncontrolled series cannot prove the advantage of aspiration over conventional surgery. Herbert et al. [17] reported a small‐sample unmatched control study (fluid aspirated from MH in 12 cases and left in situ in 8 cases). They found no significant differences of anatomical and functional outcomes between the two groups and concluded that aspiration did not appear to provide added benefit in their small sample case series [17]. In the study by Herbert et al., photoreceptors were identified in three of 12 aspirates and macrophages in three, raising concern about photoreceptor loss, although this does not establish whether the cells detached before or during aspiration [17]. Although there is a theoretical rationale for aspirating viscous intrahole fluid, its efficacy and safety compared with conventional surgery remain uncertain.

In the current study, we evaluated 101 eyes (59 eyes aspirated vs. 42 eyes nonaspirated) at 1 month postoperatively and 40 eyes (24 eyes aspirated vs. 16 eyes nonaspirated) at 3–6 months postoperatively. Our larger sample retrospective study also showed that aspiration of fluid from inside the MH did not bring significant benefits on MH closure, outer retinal restoration, and visual improvement. The MH closure rates for the two cohorts at 3–6 months postoperatively reached 58.3% and 62.5% (U‐type closure in inner retinal configuration–based classification system) and 75% and 87.5% (Type 1 closure in Kang’s classification system), respectively. The two surgical techniques showed no significant differences in terms of MH closure rate, length of EZ defect, CRT, and visual acuity improvement. Aspiration of fluid from inside the MH does not seem to bring additional benefit, and no severe damage to the macular microstructure was observed in the present cohort. After adjustment, aspiration was not significantly associated with 1‐month U‐type or Type 1 closure, or with BCVA at 1 month or 3–6 months (Table 3). Preoperative minimum hole diameter was consistently associated with closure and visual outcomes. One possible explanation for the lack of an additional benefit is that traction release and air or gas tamponade already permit fluid resorption through the RPE pump [22], so direct aspiration may add little to this process.

Improvement in the BCVA is an essential measure of the success of MH surgery. Several studies have shown the dependence of BCVA on the integrity of the outer retinal layers, particularly the EZ [23–25]. The EZ band reflects mitochondrial recovery in photoreceptors and is predictive for functional outcome [26, 27], while central foveal thickness and foveal abnormalities were not significantly correlated with final visual acuity [28]. Our cross‐sectional analysis also proved that BCVA significantly correlated with EZ defects but not with CRT at 1 month and 3–6 months postoperatively. Moreover, vision improvement was accompanied by EZ defect reduction, although the correlation between them was not so significant. Regarding the time course of EZ reconstruction, one study showed that at 1 month of closure process of MH, most eyes showed proliferating glial cells filling the foveal defect, and only 44% of eyes with glial proliferation exhibited ELM reconstruction in the fovea [29]. This indicates that at 1 month postoperatively, less than half (44%) of ELM was reconstructed, with EZ reconstruction lagging further. Another study reported that average times for postoperative recovery of the ELM and EZ were 1.5 and 6.1 months, respectively [30, 31]. These findings can explain why in our study no significant BCVA improvement occurred at 1 month post‐surgery, but significant improvement was seen at 3–6 months postoperatively. Based on OCT observations, there are two classifications of MH closure types. In 1999, Imai and colleagues [20] described U‐, V‐, and W‐type postoperative foveal contours that rely exclusively on the inner retinal profile. Subsequently, Kang and colleagues [21] proposed a classification based on the absence or presence of foveal neurosensory retinal defects. Type 1 closure may correspond to a U or V pattern, and Type 2 to a W pattern [21]. Unlike U‐type closure, where the fovea center has minimal glial cells, allowing photoreceptors almost complete reapproximation, in V‐type closure, the foveal center contains many glial cells [20]. Precisely because of these, Type 1 closure rate is higher than U‐type closure rate at each follow‐up time point, and in cross‐sectional analysis of retinal structural parameters and visual function, BCVA significantly correlated with U‐type closure but not Type 1 closure.

This study has several limitations. First, it employed a retrospective design and retrieved patient data with follow‐up information from the Hospital Information System database for analysis. Although we advised all patients to return for follow‐up at 3–6 months, many patients came from remote cities and may not have returned after learning that the MH was closed. In total, 61 of 101 patients (60.4%) had only early outcome data, and longer follow‐up is needed to determine long‐term surgical outcomes in future studies. Multivariable analyses were performed, but the small follow‐up sample limits the precision of the 3–6‐month estimates. Attrition bias is also an issue. However, we compared the baseline characteristics of patients who had follow‐up only at 1 month postoperatively with those who had continued follow‐up at 3–6 months postoperatively, and no significant differences were observed between the groups, except for the stage of MH. Specifically, 80.3% of patients followed up only at 1 month had stage B MH, compared with 50.0% of those with continued follow‐up (Supporting Table S1); this difference in morphology limits the generalizability of the later findings to the entire cohort. Second, several key steps of the MH surgery in this study were left to the surgeon’s discretion, including the choice and concentration of gas tamponade, the duration of face‐down positioning, and the decision to combine Phaco. These variabilities further compound the confounding effects introduced by surgeon‐based cohort allocation and weaken causal inference regarding the role of intrahole aspiration. The surgical skills and experience of different surgeons could also introduce bias. Given that vitreoretinal surgery outcomes are highly sensitive to subtle technical differences, the current design risks attributing surgeon‐related effects to the aspiration maneuver itself. Although all three surgeons had substantial vitreoretinal surgical experience, this does not exclude surgeon‐related differences as an explanation for the observed outcomes. Third, because the inclusion criteria were limited to primary FTMHs, the conclusions may not be generalizable to more challenging MH cases, such as recurrent or secondary MHs, or highly myopic MHs. Fourth, we employed two classification schemes to more comprehensively assess closure morphology. However, this may lead to interpretive difficulties. To minimize ambiguity, we defined “true closure” as meeting the criteria of U‐type or Type 1 closure. The proposed definition of “true closure” may not be universally accepted and should be interpreted within the context of ongoing structure–function debates in MH surgery. Fifth, further studies with adjuvant of intraoperative OCT could provide real‐time observation of the centripetal movement of the edge of MH after aspiration. Prospective randomized controlled trials with larger sample size, longer follow‐up duration, and more detailed measurements of macular anatomy and function are required to further establish the efficacy and safety of this aspiration technique.

In conclusion, in this retrospective study, compared with nonaspiration, aspiration of fluid from inside the MH seems to provide no additional benefit in the surgical treatment of medium‐to‐large primary FTMHs. The improvement of postoperative visual function occurred with the reduction of EZ defects.

## Author Contributions

**Huijin Chen**: study conception and design; **Shuang Cui** and **Dan Li**: data collection; **Changguan Wang** and **Xuefeng Feng**: data interpretation; **Ke Xu** and **Yanfeng Liao**: statistical analysis and drafting the manuscript; and **Huijin Chen**: revision of the manuscript.

**Ke Xu** and **Yanfeng Liao** contributed equally to this work and should be considered co‐first authors.

## Funding

This study was supported by Peking University‐Golden Resource “Tengyun Clinical Research Program,” Institute of Advanced Clinical Medicine, Peking University (No. TY2025005).

## Disclosure

All the authors have read and approved the final version of this manuscript.

## Ethics Statement

The studies involving human participants were reviewed and approved by the Medical Ethics Committee of Peking University Third Hospital (approval number: IRB00006761‐M20250897). Given the retrospective nature of the study, the requirement for informed consent was waived.

## Consent

Please see the Ethics Statement.

## Conflicts of Interest

The authors declare no conflicts of interest.

## Supporting Information

Additional supporting information can be found online in the Supporting Information section.

## Supporting information


**Supporting Information** Supporting Tables S1–S6. Additional analyses of follow‐up completion, 1‐month outcomes, postoperative lens‐status and minimum‐hole‐diameter subgroups, 3–6‐month outcomes, and structure–BCVA correlations.

## Data Availability

Data are available upon request from the corresponding author.
