# Supplementary material for: Internal Aspiration in Macular Hole Surgery: A Retrospective Comparative Study
Source: J Ophthalmol. 2026 Nov 1;2026:7026243. doi: 10.1155/joph/7026243 (PMC13725096; doi:10.1155/joph/7026243)
Supplement: Supplementary file 1 [file JOPH-2026-7026243-s001.docx]

**Supplementary Material for: Internal aspiration in macular hole surgery: a retrospective comparative study**

Supplementary Table S1. Baseline characteristics of eyes with or without 3–6-month follow-up

| **Parameters** | **Without 3-6-month follow-up (n=61)** | **With 3-6-month follow-up (n=40)** | **P value** |
| --- | --- | --- | --- |
| Age, years, median (Q1-Q3) | 67.00 (61.00-70.00) | 65.00 (54.00–70.00) | 0.092 |
| Symptom duration, months, median (Q1-Q3) | 6.00 (2.00-12.00) | 5.00 (1.00–12.00) | 0.787 |
| BCVA, logMAR, median (Q1-Q3) | 1.00 (0.92-1.30) | 1.00 (0.92-1.40) | 0.569 |
| IOP (mmHg) | 13.80 ± 2.59 | 15.00 (12.00-17.00) | 0.061 |
| Minimum diameter, μm | 480.00 (370.00–613.50) | 521.50 (318.75-607.58) | 0.586 |
| Basal diameter, μm | 996.71 ± 258.62 | 1058.22±367.94 | 0.326 |
| Gender (female), n (%) | 46 (75.4%) | 27 (67.5%) | 0.385 |
| Stage of the MH, n (%) |  |  | 0.003 |
| Stage A | 1 (1.6%) | 2 (5.0%) |  |
| Stage B | 49 (80.3%) | 20 (50.0%) |  |
| Stage C | 11 (18.0%) | 15 (37.5%) |  |
| Stage D | 0 (0.0%) | 3 (7.5%) |  |
| With ERM, n (%) | 18 (29.5%) | 12 (30.0%) | 0.958 |
| With operculum, n (%) | 22 (36.1%) | 17 (42.5%) | 0.516 |
| Posterior vitreous detachment, n (%) |  |  | 0.253 |
| Not detected | 33 (54.1%) | 16 (40.0%) |  |
| Complete PVD | 23 (37.7%) | 17 (42.5%) |  |
| Persistent vitreous adhesion | 5 (8.2%) | 7 (17.5%) |  |
| Preoperative lens status, n (%) |  |  | 0.599 |
| Clear crystalline lens | 5 (8.2%) | 6 (15.0%) |  |
| Cataract | 52 (85.2%) | 32 (80.0%) |  |
| Pseudophakia | 4 (6.6%) | 2 (5.0%) |  |
| Combined Phaco+IOL, n (%) | 52 (85.2%) | 30 (75.0%) | 0.198 |
| Choice of tamponade, n (%) |  |  | 0.307 |
| Sterile air | 35 (57.4%) | 27 (67.5%) |  |
| C3F8 gas | 26 (42.6%) | 13 (32.5%) |  |
| Treatment allocation (aspiration), n (%) | 35 (57.4%) | 24 (60.0%) | 0.794 |
| Surgeon, n (%) |  |  | 0.965 |
| Doctor A | 26 (42.6%) | 18 (45.0%) |  |
| Doctor B | 9 (14.8%) | 6 (15.0%) |  |
| Doctor C | 26 (42.6%) | 16 (40.0%) |  |

Data are presented as mean ± SD, median (Q1-Q3), or n (%), as appropriate. Continuous variables were compared using Student's t-tests when both groups met normality assumptions and Mann-Whitney U tests otherwise. Pearson χ² tests (without continuity correction) were used when all expected cell frequencies were ≥5; otherwise, Fisher exact tests were used. BCVA, best-corrected visual acuity; C3F8, perfluoropropane; ERM, epiretinal membrane; IOL, intraocular lens; IOP, intraocular pressure; PVD, posterior vitreous detachment; SD, standard deviation.

Supplementary Table S2. Clinical outcomes of the 101 eyes with follow-up at 1 month postoperatively

|  | **Cohort 1 (n=59)** | **Cohort 2 (n=42)** | ***P*-value** |
| --- | --- | --- | --- |
| BCVA(LogMAR),  median (q1-q3) | 1 (0.82-1.30） | 0.96 (0.82-1.30） | 0.809 |
| Inner retinal configuration-based classification, n (%) |  |  |  |
| U-type closure | 29 (49.2%) | 22 (52.4%) | 0.749 |
| V- type closure | 19 (32.2%) | 13 (31%) |  |
| W- type closure | 11 (18.6%) | 7 (16.7%) |  |
| Kang’s classification, n (%) |  |  |  |
| Type 1 closure | 48 (81.4%) | 35 (83.3%) | 0.798 |
| Type 2 closure | 7 (11.9%) | 7 (16.7%) |  |
| Type 3 closure | 4 (6.8%) | 0 |  |
| CRT (μm), median (q1-q3) | 261.00 (227.00-303.00) | 277.00 (261.00-307.60) | 0.121 |
| EZ defect (μm), median (q1-q3) | 1334.00 (900.75-1777.25) | 1420.00 (1107.00-1766.00) | 0.535 |
| Reoperation for unclosed hole, n (%) | 4 (6.8%) | 3 (7.1%) | 1.000 |

CRT, central retinal thickness; EZ, ellipsoid zone.

Supplementary Table S3. Best-corrected visual acuity stratified by postoperative lens status

**A. Between-cohort comparisons**

| **Analysis population and time point** | **Pseudophakic eyes** | | | **Phakic eyes** | | |
| --- | --- | --- | --- | --- | --- | --- |
|  | **Cohort 1** | **Cohort 2** | **P value** | **Cohort 1** | **Cohort 2** | **P value** |
| All 101 eyes, 1 month | 1.00 (0.82-1.28) | 0.96 (0.82-1.28) | 0.740 | 0.92 ± 0.28 | 0.96 (0.84-1.30) | 0.596 |
| 40-eye follow-up subset, 1 month | 1.00 (0.82-1.30) | 1.30 (1.01-2.10) | 0.111 | 0.92 ± 0.39 | 1.30 (0.92-1.30) | 0.442 |
| 40-eye follow-up subset, 3–6-months | 0.82 (0.52-1.00) | 0.83 ± 0.39 | 0.889 | 0.92 (0.61-1.01) | 0.87 ± 0.52 | 0.880 |

**B. Within-cohort paired comparisons: pseudophakic eyes**

| **Analysis population and comparison** | **Cohort 1** | | | **Cohort 2** | | |
| --- | --- | --- | --- | --- | --- | --- |
|  | **First time point** | **Second time point** | **P value** | **First time point** | **Second time point** | **P value** |
| All 101 eyes: preoperative vs 1 month | 1.10 (1.00-1.40) | 1.00 (0.82-1.28) | 0.026 | 1.00 (0.85-1.23) | 0.96 (0.82-1.28) | 0.681 |
| 40-eye follow-up subset: preoperative vs 1 month | 1.00 (0.92-1.40) | 1.00 (0.82-1.30) | 0.276 | 1.00 (0.87-1.30) | 1.30 (1.01-2.10) | 0.286 |
| 40-eye follow-up subset: 1 month vs 3–6-months | 1.00 (0.82-1.30) | 0.82 (0.52-1.00) | 0.003 | 1.30 (1.01-2.10) | 0.83 ± 0.39 | 0.009 |

**C. Within-cohort paired comparisons: phakic eyes**

| **Analysis population and comparison** | **Cohort 1** | | | **Cohort 2** | | |
| --- | --- | --- | --- | --- | --- | --- |
|  | **First time point** | **Second time point** | **P value** | **First time point** | **Second time point** | **P value** |
| All 101 eyes: preoperative vs 1 month | 1.00 (1.00-1.10) | 0.92 ± 0.28 | 0.419 | 1.05 (0.87-1.80) | 0.96 (0.84-1.30) | 0.624 |
| 40-eye follow-up subset: preoperative vs 1 month | 1.00 (1.00-1.05) | 0.92 ± 0.39 | 0.789 | 1.10 (1.00-1.40) | 1.30 (0.92-1.30) | 0.787 |
| 40-eye follow-up subset: 1 month vs 3–6-months | 0.92 ± 0.39 | 0.92 (0.61-1.01) | 0.371 | 1.30 (0.92-1.30) | 0.87 ± 0.52 | 0.423 |

BCVA values are presented as mean ± SD or median (Q1-Q3) in logMAR units. Between-cohort P values were calculated using the Mann-Whitney U test; paired P values were calculated using the Wilcoxon signed-rank test. Pseudophakic eyes comprised eyes with baseline pseudophakia or combined Phaco+IOL. BCVA, best-corrected visual acuity; IOL, intraocular lens; SD, standard deviation.

Supplementary Table S4. Macular hole closure stratified by preoperative minimum hole diameter

**A. Large holes (>400 μm)**

| **Closure pattern** | **Cohort 1** | **Cohort 2** | **Risk difference (95% CI), %** | **P value** |
| --- | --- | --- | --- | --- |
| **All 101 eyes, 1 month** | | | | |
| U-type closure | 17/42 (40.5%) | 11/27 (40.7%) | -0.3% (-24.0, 23.5) | 0.983 |
| Type 1 closure | 31/42 (73.8%) | 21/27 (77.8%) | -4.0% (-24.5, 16.6) | 0.709 |
| **40-eye follow-up subset, 1 month** | | | | |
| U-type closure | 5/17 (29.4%) | 2/10 (20.0%) | 9.4% (-23.5, 42.3) | 0.678 |
| Type 1 closure | 10/17 (58.8%) | 7/10 (70.0%) | -11.2% (-48.0, 25.6) | 0.692 |
| 40-eye follow-up subset, 3–6-months | | | | |
| U-type closure | 8/17 (47.1%) | 4/10 (40.0%) | 7.1% (-31.5, 45.6) | 1.000 |
| Type 1 closure | 11/17 (64.7%) | 8/10 (80.0%) | -15.3% (-48.9, 18.3) | 0.666 |

**B. Medium holes (250–400 μm)**

| **Closure pattern** | **Cohort 1** | **Cohort 2** | **Risk difference (95% CI), %** | **P value** |
| --- | --- | --- | --- | --- |
| **All 101 eyes, 1 month** | | | | |
| U-type closure | 12/17 (70.6%) | 11/15 (73.3%) | -2.7% (-33.9, 28.4) | 1.000 |
| Type 1 closure | 17/17 (100.0%) | 14/15 (93.3%) | 6.7% (-6.0, 19.3) | 0.469 |
| **40-eye follow-up subset, 1 month** | | | | |
| U-type closure | 5/7 (71.4%) | 6/6 (100.0%) | -28.6% (-62.0, 4.9) | 0.462 |
| Type 1 closure | 7/7 (100.0%) | 6/6 (100.0%) | — | — |
| 40-eye follow-up subset, 3–6-months | | | | |
| U-type closure | 6/7 (85.7%) | 6/6 (100.0%) | -14.3% (-40.2, 11.6) | 1.000 |
| Type 1 closure | 7/7 (100.0%) | 6/6 (100.0%) | — | — |

Data are presented as n/N (%). Risk difference was calculated as Cohort 1 minus Cohort 2. Pearson χ² tests (without continuity correction) were used when all expected cell frequencies were ≥5; otherwise, Fisher exact tests were used. Rows with no outcome variation were not tested. No interaction test was performed, and the results do not establish effect modification. CI, confidence interval.

Supplementary Table S5. Clinical outcomes of the 40 eyes with follow-up at both 1 month and 3–6 months postoperatively

|  | **1 month postoperatively** | | | **3–6 months postoperatively** | | |
| --- | --- | --- | --- | --- | --- | --- |
|  | **Cohort 1(n=24)** | **Cohort 2(n=16)** | ***P*-value** | **Cohort 1(n=24)** | **Cohort 2(n=16)** | ***P*-value** |
| BCVA(LogMAR), median (q1-q3) | 1.00, (0.82-1.30) | 1.30, (0.92-2.30) | 0.101 | 0.82, (0.52-1.00) | 0.82, (0.54-1.23) | 0.902 |
| Inner retinal configuration-based classification, n (%) |  |  |  |  |  |  |
| U-type closure | 10(41.7%) | 8(50.0%) | 0.604 | 14(58.3%) | 10(62.5%) | 0.792 |
| V- type closure | 7(29.2%) | 5(31.3%) |  | 4(16.7%) | 4(25.0%) |  |
| W- type closure | 7(29.2%) | 3(18.8%) |  | 6(25%) | 2(12.5%) |  |
| Kang’s classification, n (%) |  |  |  |  |  |  |
| Type 1 closure | 17(70.8%) | 13(81.3%) | 0.450 | 18(75%) | 14(87.5%) | 0.321 |
| Type 2 closure | 4(16.7%) | 3(18.8%) |  | 5(20.8%) | 2(12.5%) |  |
| Type 3 closure | 3(12.5%) | 0 |  | 1(4.2%) | 0 |  |
| CRT(μm), median (q1-q3) | 258.50(206.50-314.25) | 274.00(253.00-311.00) | 0.283 | 242.50(196.75-287.25) | 250.00(230.00-287.00) | 0.636 |
| EZ defect(μm), median (q1-q3) | 1441.00(1064.00-1749.00) | 1375.50(1071.75-1879.75) | 0.960 | 750.50(303.75-1709.50) | 564.00(40.00-1251.00) | 0.243 |
| Reoperation for unclosed hole, n (%) | 3(12.5%) | 2(12.5%) | 1.000 | 0 | 0 |  |

CRT, central retinal thickness; EZ, ellipsoid zone.

Supplementary Table S6. Cross-sectional analysis of foveal microstructural parameters and visual function in 40 patients with follow-up at 1 month and 3–6 months postoperatively

|  | **1 month postoperatively** | | **3-6 months postoperatively** | |
| --- | --- | --- | --- | --- |
|  | **Spearman Rho** | **P value** | **Spearman Rho** | **P value** |
| U-type closure | -0.481 | 0.002 | -0.342 | 0.031 |
| Type 1 closure | -0.241 | 0.134 | -0.198 | 0.220 |
| CRT (μm) | -0.134 | 0.418 | -0.264 | 0.115 |
| EZ defect (μm) | 0.402 | 0.015 | 0.579 | <0.001 |

CRT, central retinal thickness; EZ, ellipsoid zone.
